# Ultrasonography (US) in the assessment of pediatric non traumatic gastrointestinal emergencies

**DOI:** 10.1186/2036-7902-5-S1-S12

**Published:** 2013-07-15

**Authors:** Paolo Fonio, Francesco Coppolino, Anna Russo, Alfredo D’Andrea, Antonella Giannattasio, Alfonso Reginelli, Roberto Grassi, Eugenio Annibale Genovese

**Affiliations:** 1 University of Turin, Institute of Diagnostic and Interventional Radiology, Turin, Italy; 2 University of Palermo, Department of Radiology, Palermo, Italy; 3 S G. Moscati Hospital, Department of Radiology, Aversa, Italy; 4 University of Molise, Department of Health and Science, Campobasso, Italy; 5 Second University of Naples, Department of Clinical and Experimental Internistic F. Magrassi, Naples, Italy; 6 University of Cagliari, Department of Radiology, Cagliari, Italy

**Keywords:** gastrointestinal pediatric emergencies, intussusceptions, appendicitis, hypertrophic pyloric stenosis, volvulus

## Abstract

**Background:**

Non traumatic gastrointestinal emergencies in the children and neonatal patient is a dilemma for the radiologist in the emergencies room and they presenting characteristics ultrasound features on the longitudinal and axial axis. The most frequent emergencies are : appendicitis, intussusceptions, hypertrophic pyloric stenosis, volvulus due to intestinal malrotation. The aim of this article is to familiarize the reader with the US features.

**Methods:**

A retrospective analysis of 200 ultrasound examinations performed in neonatal and children patients with fever, abdominal pain, leukocytosis, vomiting and diarrhea were evaluated.

**Results:**

Of 200 exame 50 cases of intussusceptions, 100 cases of appendicitis, 20 cases associated with abscess;10 gangrenous appendicitis with absence a color Doppler , and 10 cases of perforated appendicitis at tomography computer integration and 10 cases of volvulus was found.

**Conclusions:**

Ultrasonography (US) is therefore rapidly becoming an important imaging modality for the evaluation of acute abdominal pain, particularly in pediatric patients, where satisfactory examination is often not achievable for the attending clinicians. US provides excellent anatomic detail on the longitudinally and axial axis .

## Background

Non traumatic gastrointestinal emergencies in the children and neonatal patient is a dilemma for the radiologist in the emergencies room, they presenting characteristics ultrasound features on the longitudinal and axial axis .The abdominal pain in children is one of the most common presentations at the emergency room. Inability to give reliable history, atypical clinical presentations, numerous extra-abdominal causes and the painful abdomen in children often causes difficulty in arriving at the correct diagnosis and causing diagnostic dilemma. Early diagnosis is the first step towards proper management of a patient presenting with acute abdomen and pain. The radiologist, therefore, plays a key role in the initial care of these emergencies pathology in infants and the appropriate surgical referral. It is important that the radiologist understand the anatomic changes in affected infants as reflected by imaging techniques.

The objective of this article is to familiarize the reader with the US features of the most common and some of the least common non traumatic gastrointestinal emergencies. This is a retrospective analysis of 200 ultrasound examinations performed in neonatal and the children patient arriving in the emergency room with fever, abdominal pain, leukocytosis, vomiting and diarrhea. B-mode and Color Doppler ultrasonography has become the imaging modality of choice for evaluating non traumatic gastrointestinal emergencies. In the present study, we have discussed the diagnostic efficacy of ultrasonography in the evaluation of acute abdominal conditions in pediatric age group, and we have presented the tipical features on the longitudinal and axial axis

## Methods

We evaluated retrospectively 200 ultrasound exams effectuated between January 2009 and December 2011, in 200 patients aged few day to 14 years old arrived in emergency room for abdominal pain, vomiting, leukocytosis, fever and diarrhea. Ultrasonography (US) was done using 3.5 MHz and 7.5 MHz transducers and the image are examinated effectuated on the longitudinally axis and cross axis combined with colour Doppler ultrasonography (CDUS). At the start of the examination, the patient is asked to point to the site of maximal tenderness.

## Results and discussion

We found 50 cases of intussusception. On the axial scans we evaluated “crescent in doughnut sign” and “concentric ring sign”, a sign of the donut. On the longitudinal scan the “ sandwich sign”, and “hairfork sign”. We found 100 cases of appendicitis, 20 cases associated with abscess;10 gangrenous appendicitis with absence a color Doppler , and 10 cases of perforated appendicitis at computer tomography integration.The ultrasonographic sign in axial scans is “target sign”, in longitudinal scans: a fluid-filled uncompressible, blind-ending tubular structure; parietal diameter> or = 6mm. We found 20 cases of hypertrophic pyloric stenosis. The sonographic features on the axial scans the is “donut sign”. On the longitudinal scans is “double track sign”

We found 10 cases of volvulus. On the axial scan we found “whirlpool sign” formed by the mesenteric vein is on the left side of the artery, and the arterio-venous dilatation of the loop upstream; hyper-echoic thickening of the bowel wall edema; course spiral loop.

In our study we found 50 cases of intussusception. Intussusception is one the most common causes of the acute abdomen in infancy [1]. Intussusception occur when a portion of the digestive tract becomes telescoped into the adjacent bowel segment. Generally occurs in children between 6 month and 2 year age. The vast majority are ileocolic [2,3].

In our experience the mean age is 1 year, and the tract of the intussusception are ileum-colic in all patient. 30 patient had a classic clinical triad :currant-jelly stools, hematochezia and a palpable mass, 5 patients had a convulsion after vomiting and 15 patient had a vomiting.

Ultrasound is a highly accurate in the diagnosis with a sensitivity of 98%-100% and a specificity of 88%-100% [1] .

The component of intussusception produced characteristics sign on the ultrasonography scan. This sign are on the longitudinally axis “sandwich sign” and “hair-fork sign” and on the axial axis are “crescent in donught sign” and “concentric ring sign”.

In US modality the intussusceptions had a large mass, usually greater than 5x2.5cm, that often displaces adjacent bowel loops[4]. In our study this mass is palpable in 35 patients and is localized on the sub-hepatic region.

**Figure 1:**
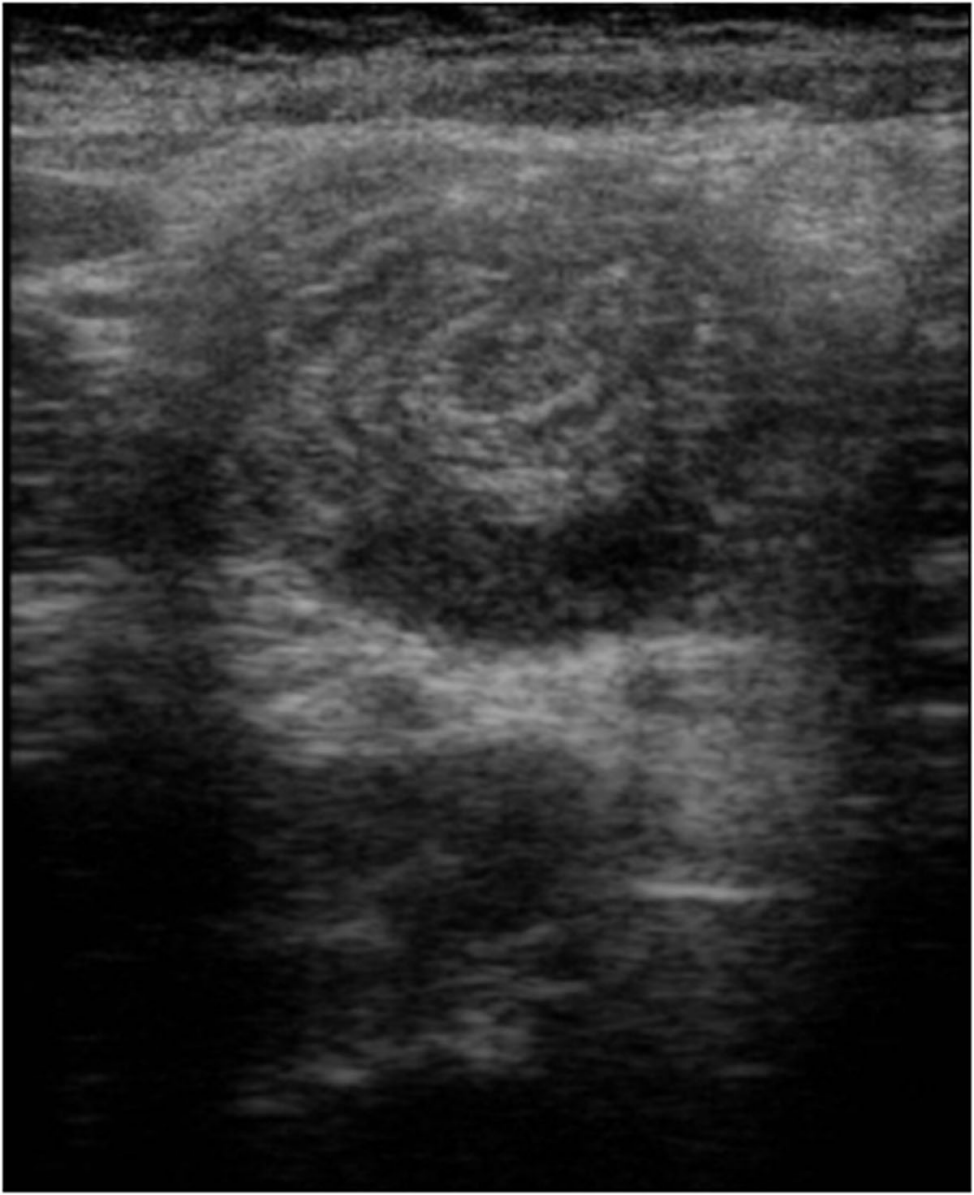
Ultrasound features of intussusceptions. US obtained at the apex shows a hyper echoic outer ring separated from a hypo echoic center by a thin hyper echoic ring, which likely represents the opposed serous surface of the intussuscepted.

**Figure 2:**
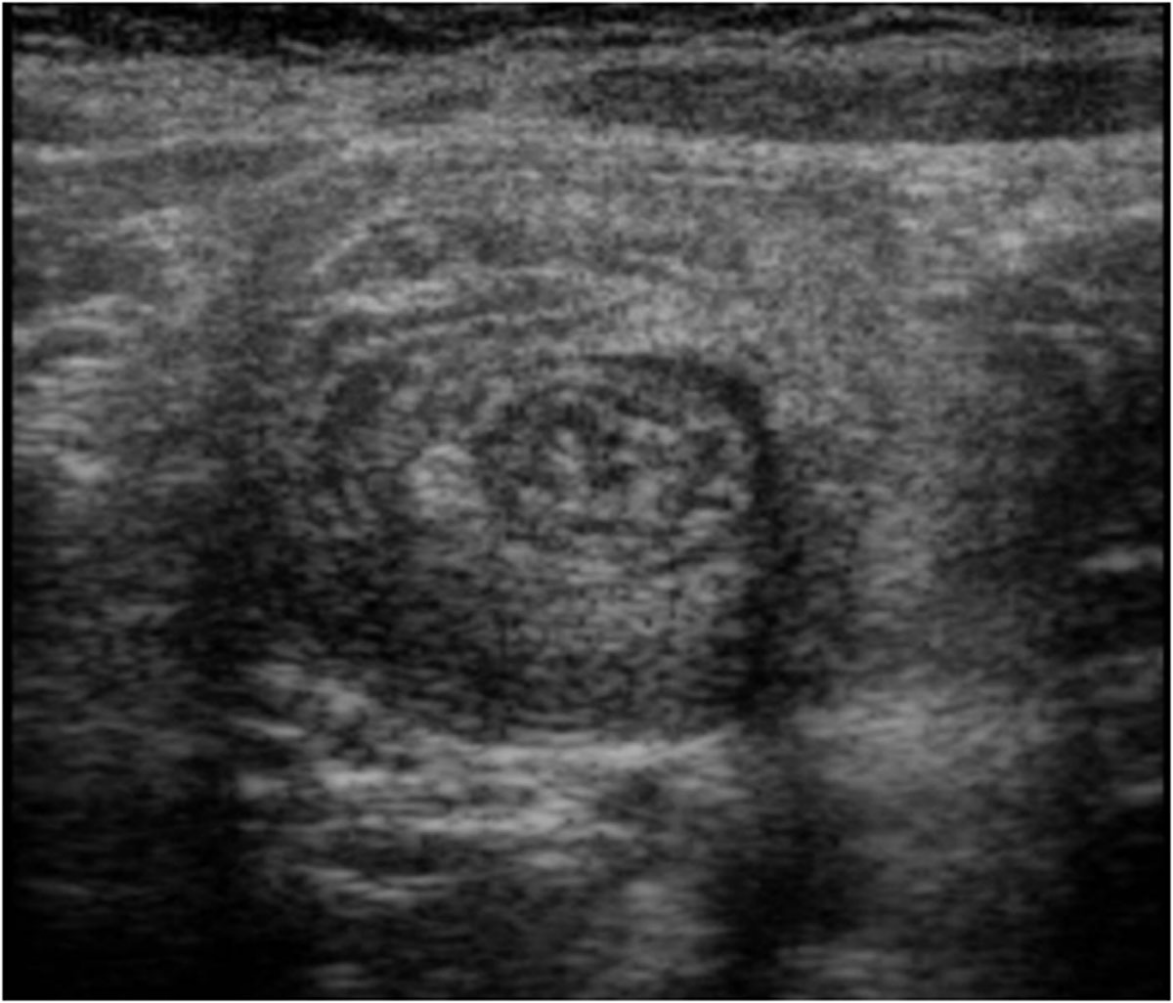
Ultrasound features of intussusceptions. Us obtained near the apex shows multiple concentric rings with a hypo-echoic ring surrounding a hyper-echoic ring, which surrounds another hypo echoic ring.

**Figure 3:**
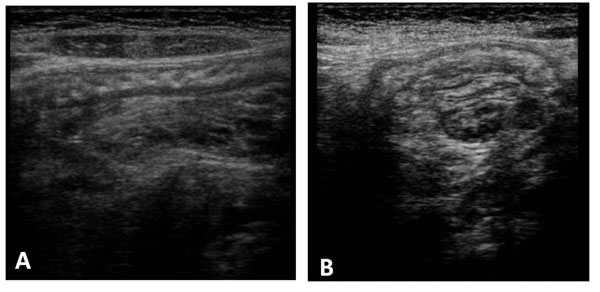
Ultrasound features of intussusceptions. On the longitudinal scan obtained at the centre of the mass show the sandwich sign formed by three parallel hypo-echoic bands separated by two nearly parallel hyper-echoic bands. The outer hypo-echoic band represent the edematous everted limb of the intussuscepted and the thin intussusceptions, the central hypo-echoic band is the central limb of the intussuscepted. The hyper-echoic bands are caused by the mesentery that is dragged along with the bowel loop. Us scan obtained at the apex show the hair-fork-sign form by three parallel hypo- echoic bands (the prongs of the hair fork), separated by two hyper-echoic bands formed by mesentery. (a) Us scan obtained at the base shows the central limb of the intussusceptions eccentrically surround by the hyper-echoic mesentery that show the crescent in doughnut sign. (b)

On the US image the intussusceptions is a complex structure. The intussusceptions (the receiving loop) contains the folded intussuscepted (the donor loop), which has two components: the entering limb and returning limbs. On axial intussusception has a variable appearance, which is primarily due to the amount of mesentery[5]. Us obtained at the apex shows a hyper echoic outer ring separated from a hypo echoic center by a thin hyper echoic ring, which likely represents the opposed serous surface of the intussuscepted. (Fig. 1) Us obtained near the apex shows multiple concentric rings with a hypo-echoic ring surrounding a hyper-echoic ring, which surrounds another hypo echoic ring. (Fig. 2) Us scan obtained at the base shows the central limb of the intussusception eccentrically surround by the hyper-echoic mesentery that show the crescent in doughnut sign.(Fig. 3a)

On the longitudinal scan obtained at the centre of the mass show the sandwich sign formed by three parallel hypo-echoic bands separated by two nearly parallel hyper-echoic bands. (Fig. 3b) The outer hypo-echoic band represent the edematous everted limb of the intussuscepted and the thin intussusceptions, the central hypo-echoic band is the central limb of the intussuscepted. The hyper-echoic bands are caused by the mesentery that is dragged along with the bowel loop. Us scan obtained at the apex show the hair-fork-sign form by three parallel hypo- echoic bands (the prongs of the hair fork), separated by two hyper-echoic bands formed by mesentery[1-6]. Another sign on the longitudinal scan is “pseudo-kidney sign” is rare and is visualized when the intussusception is curved or is imaged obliquely and the mesentery is demonstrated on only one side of the central limb of the intussuscepted [7].

We found 100 cases of acute appendicitis.Acute appendicitis is one of the major causes of hospitalization in children. Acute appendicitis is the most common condition requiring emergency abdominal surgery in the pediatric population, with 60,000–80,000 cases annually in the UnitedStates [8,9].The condition typically develops in older children and young adults. It is rare under the age of 2 years. The lifetime risk of acute appendicitis ranges from 7% to 9% [10]. Acute appendicitis presents a challenging problem to caregivers because it must be differentiated from a variety of other conditions that result in acute abdominal pain in childhood and the most common condition requiring emergency abdominal surgery in the pediatric population. In the our experience 80 patients had crampy, per-umbilical or right lower quadrant pain; nausea; vomit; point tenderness in the right lower quadrant; rebound tenderness; and leukocytosis with a left shift. 20 patient had fever and vomit, and 20 patients had tenderness in the right lower and leukocytosis.

Although knowledge of the classic findings is important, the clinical diagnosis of acute appendicitis in children is not always straightforward. Us is a highly accurate in the diagnosis with a sensitivity of 44%-94% and a specificity of 47%-95% [8].

**Figure 4:**
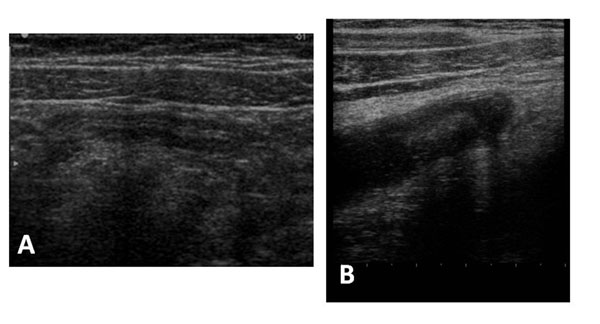
Ultrasound features of appendicitis. On longitudinal images, the inflamed, non perforated appendix appears as a fluid-filled, uncompressible, blind-ending tubular structure. The maximal appendix diameter, from outside wall to outside wall, is greater than 6 mm. (a) In early non perforated appendicitis, on the longitudinal images an inner echogenic lining representing sub-mucosa can be identified.(b)

**Figure 5:**
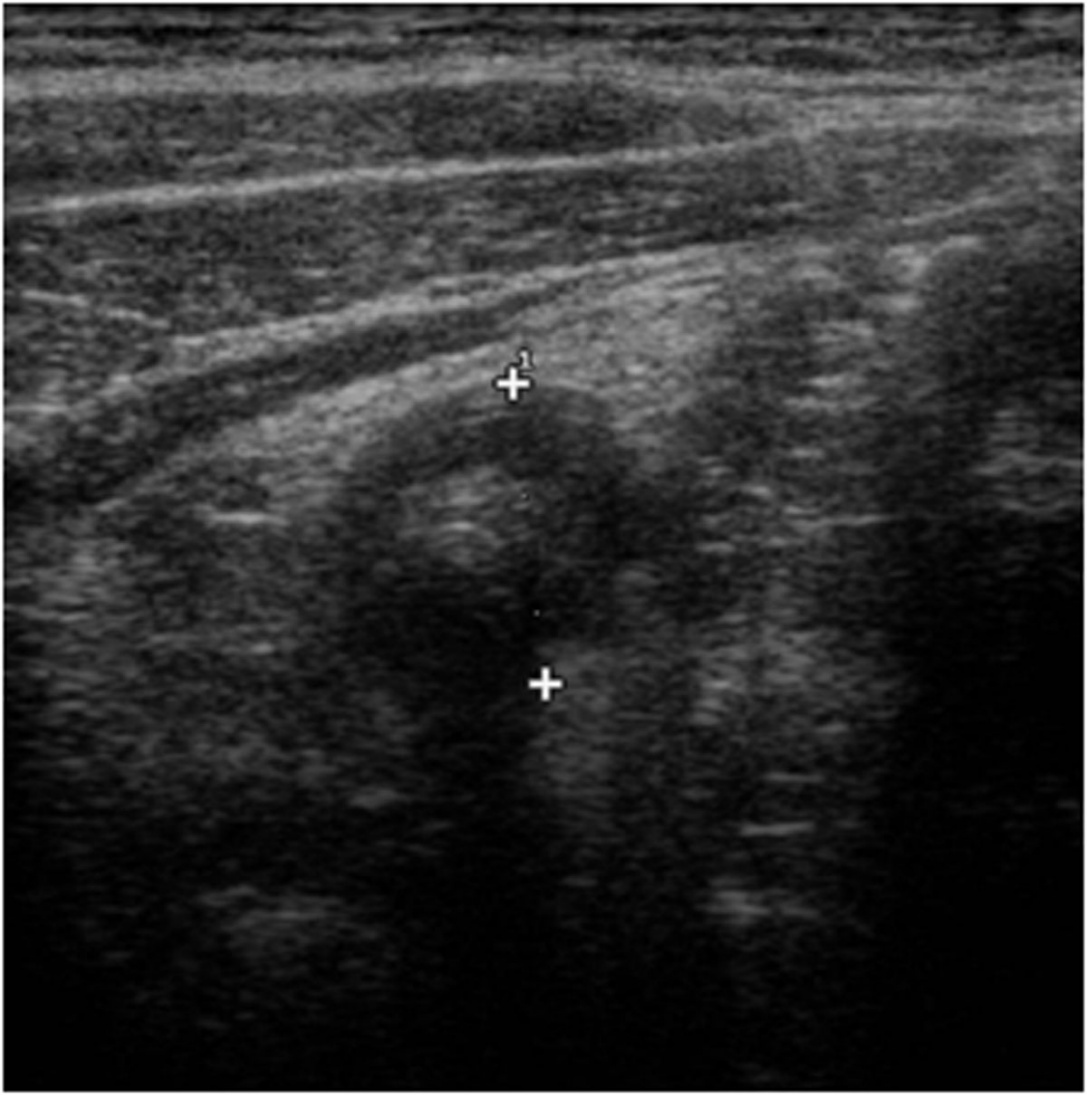
Ultrasound features of appendicitis. On the axial image, we valuated a “target sign” characterized by a fluid-filled center and surrounded by a echogenic mucosa and sub-mucosa and hypo-echoic muscularis.

We performed the graded-compression technique of US with linear array transducer. Gentle, gradual pressure we used to compress the anterior abdominal wall, resulting in displacement and compression of normal bowel loops. Adequate compression has been achieved if the iliac vessels and psoas muscle are visualized, since the appendix will be anterior to these structures. Scanning is performed in both longitudinal and transverse planes, and the examination begins with identification of the ascending colon, which appears as a non peristaltic structure containing gas and fluid. The transducer is then moved inferiorly to identify the terminal ileum, which is easily compressible and displays active peristalsis. This is useful to expedite the examination and to aid in locating a retro-caecum appendix. On longitudinal images, the inflamed, non perforated appendix appears as a fluid-filled, uncompressible, blind-ending tubular structure. The maximal appendix diameter, from outside wall to outside wall, is greater than 6 mm. (Fig. 4a) In early non perforated appendicitis, on the longitudinal images an inner echogenic lining representing sub-mucosa can be identified. (Fig. 4b) On the axial image, we evaluated a “target sign” characterized by a fluid-filled center and surrounded by a echogenic mucosa and sub-mucosa and hypo-echoic muscularis [9,10]. (Fig. 5)

**Figure 6:**
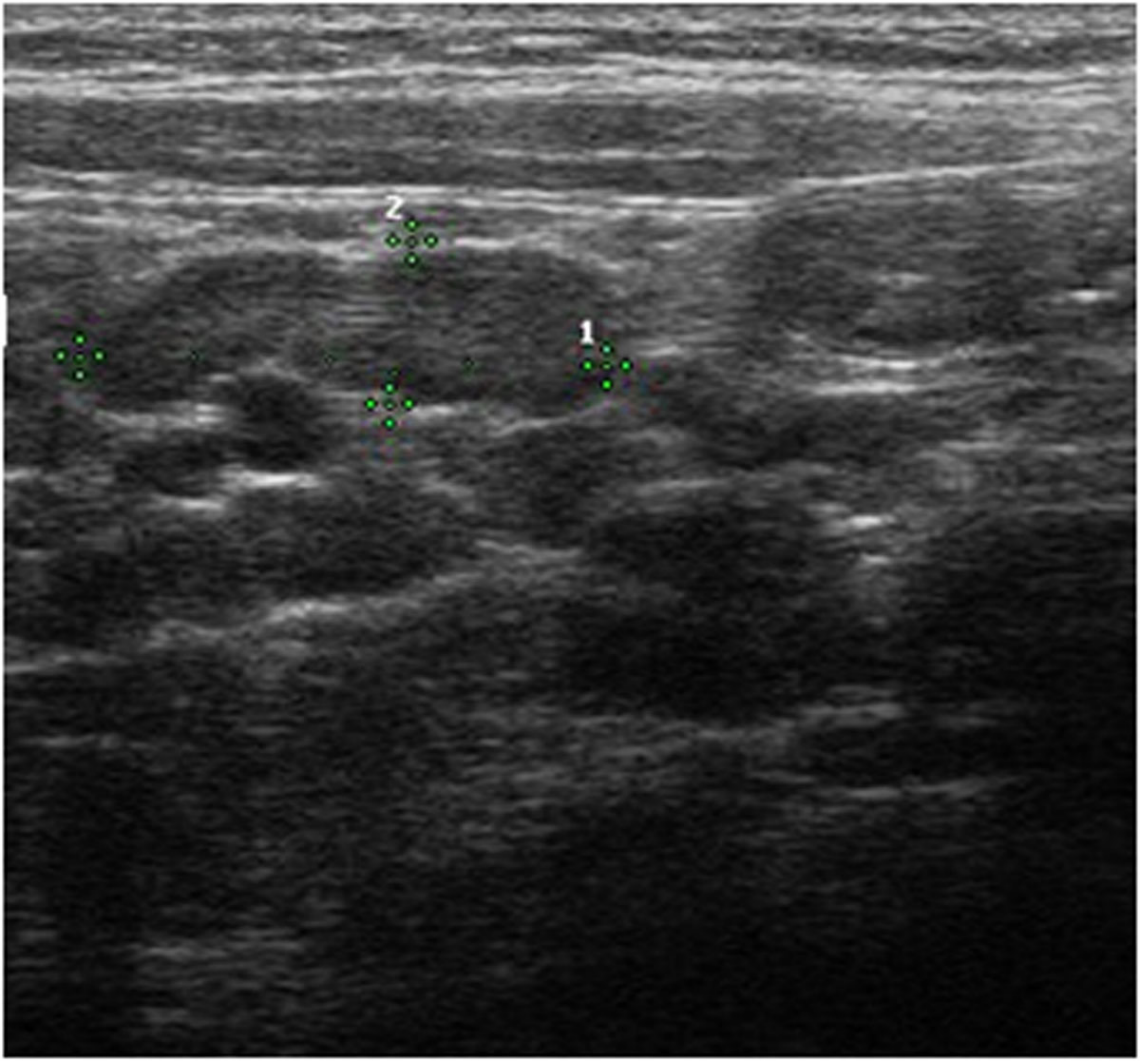
Ultrasound features of appendicitis. Other findings of appendicitis include enlarged mesenteric lymph nodes.

Other findings of appendicitis include on the longitudinal axis and on the axial axis an appendicolith, which appears as an echogenic foci with acoustic shadowing ; peri-cecal or periappendicular fluid; increased periappendicular echogenicity representing fat infiltration ; and enlarged mesenteric lymph nodes. (Fig. 6)

US features of perforation include loss of the echogenic sub-mucosal layer and presence of a loculated periappendicular or pelvic fluid collection or abscess In the our experience we found 20 cases of abscess;10 gangrenous appendicitis with absence a color Doppler , and 10 cases of perforated appendicitis at computer tomography integration [11-13]. We use color Doppler US because provides a useful adjunct in the evaluation of suspected acute appendicitis[14,15]. Color Doppler US of non perforated appendicitis typically demonstrates peripheral wall hyperemia, reflecting inflammatory hyper-perfusion [16-18]. In early inflammation, color flow may be absent or limited to the appendicular tip. Color flow may also be absent in gangrenous appendicitis. Color Doppler findings of appendicular perforation include hyperemia in the periappendicular soft tissues or within a well-defined abscess .

We found 20 cases of hypertrophic pyloric stenosis (HPS), mean age seven week of life. Infantile hypertrophic pyloric stenosis is a common condition affecting young infants in the fourth-seven week of life; despite its frequency, it has been recognized only for a little over a century, and its etiology remains unknown. The male-to-female ratio is approximately 4:1, with reported ratios ranging from 2.5:1 to 5.5:1 [19].

The lesion is characterized by gastric outlet obstruction and multiple anatomic abnormalities of the pyloric antrum. Typically, infants with HPS are clinically normal at birth; during the first few weeks of postnatal life, they develop non bilious forceful vomiting described as “projectile.” The clinical diagnosis hinges on palpation of the thickened pylorus, or “olive” [20]. In our experience the patients had vomiting and only 10 presented a olive palpable.

Ultrasonography exam of the antrum-pyloric region were obtained in all patient: first, with the infant in supine position; and then, with the patient in the lateral decubitus position, right side down . In the supine position, we effectuated axial scanning . In the decubitus position on the right side, we effectuated a longitudinal scanning [21]. If sufficient fluid was not present in the stomach to outline the antrum-pyloric region, the infant was given 1 to 2 ounces of sugar water or milk orally and we observe visually the passage of gastric content through the pyloric canal into the duodenum.

In the normal pylor when the canal is viewed in cross section we evaluated the “bulls-eye” sign performed by outer anechoic rim, which is almost too thin to measure, represents the normal circular (Torgensen’s) muscle, and he inner echogenic layer represents the mucosa and submucosa. The innermost anechoic center represents fluid in the pyloric canal**.** In the normal anatomy of pylorus the transverse diameter is 0.7-1.1 cm , the length of pyloric canal is 1.0-1.3 cm and the thickness of circular muscle is too thin to measure [22].

**Figure 7:**
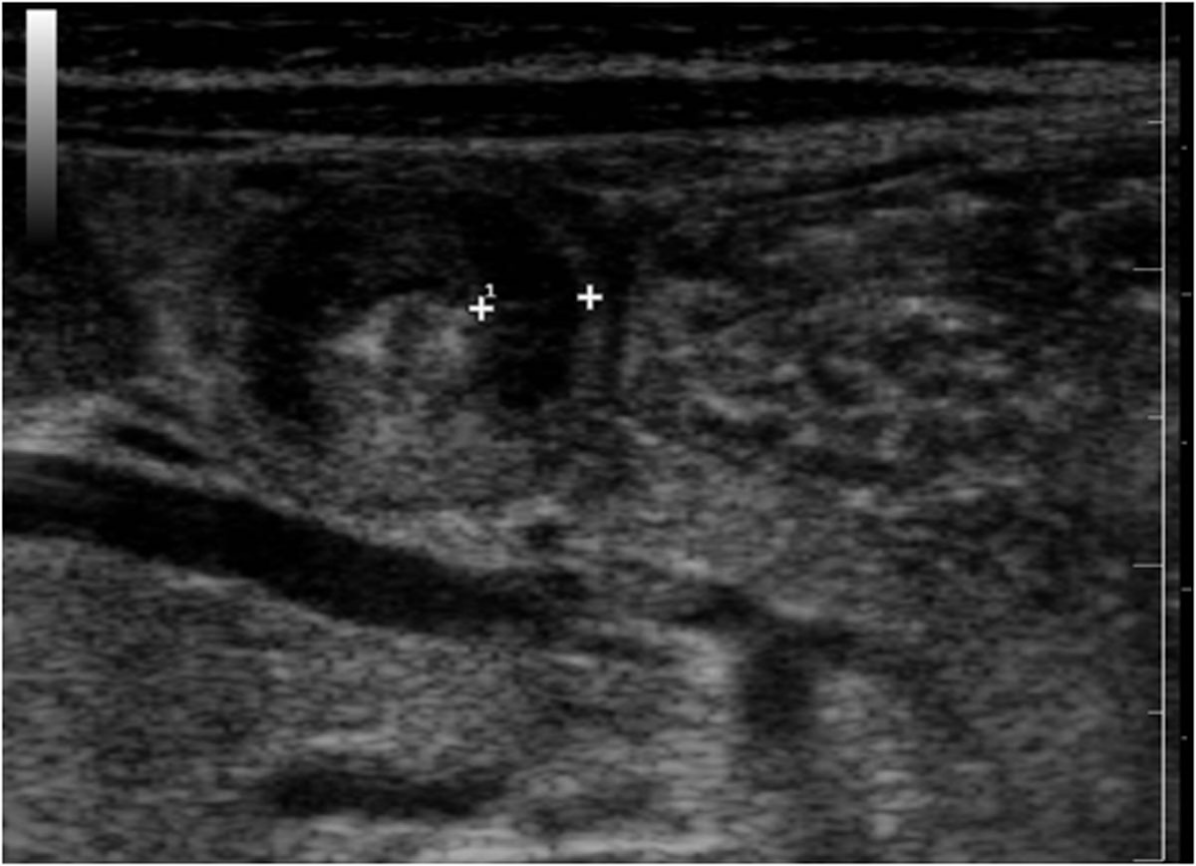
Ultrasound features of hypertrophic pyloric stenosis. On the cross scanning with the infant in the supine position is visualized the “donut sign” consists of a prominent anechoic rim of thickened muscle, and an echogenic center of mucosa and submucosa .

In the hypertrophic stenosis on the cross scanning with the infant in the supine position is visualized the “donut sign” consists of a prominent anechoic rim of thickened muscle, and an echogenic center of mucosa and submucosa [23] (Fig. 7).On this scan the transverse diameter is measured and in our experience mean 1.3cm, and the thickness of circular muscle is >0.4-1cm.

In the longitudinal plane with the patient in the lateral decubitus position, right side down we observed the continuity between anechoic rim of hypertrophied circular muscle and the normal muscle of the gastric antrum . In this plane, by changing the degree of obliquity of the patient, can demonstrate the same anatomic features of the canal in the longitudinal plane **,** and can visualize peristalsis; and can evaluate the thickness of the muscle and the overall length of the pyloric canal. On this view hypertrophic muscle remains closed, and no fluid passes into the duodenum. On the longitudinally view we evaluate the “nipple sign”, characterized of the muscle hypertrophic to a variable degree, mucosa crowded, thickened to a variable degree, and protrusion into the distended portion of the antrum [23-25].

**Figure 8:**
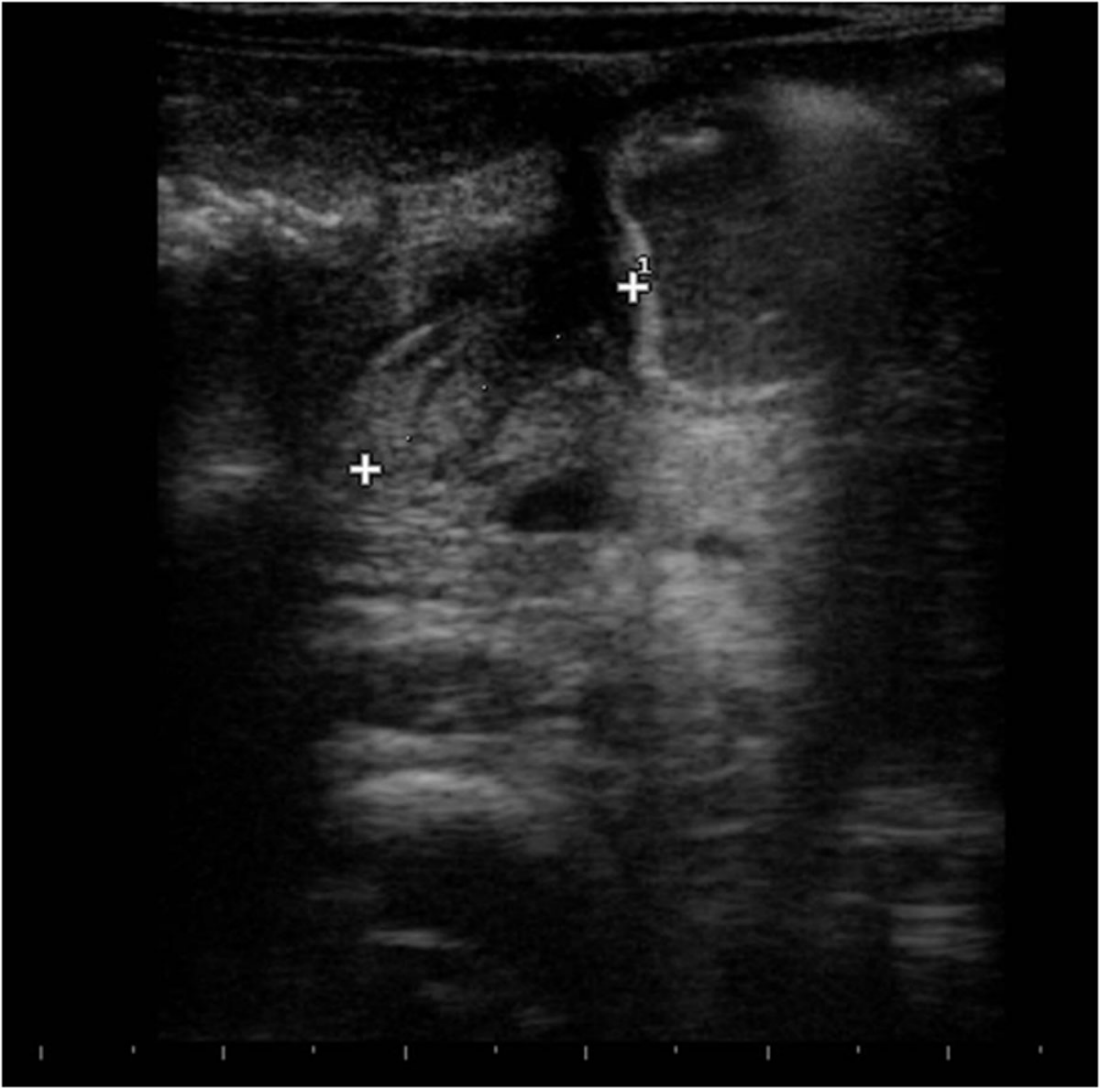
Ultrasound features of hypertrophic pyloric stenosis. On this view, we evaluate the “double track sign” characterized by double layer of redundant mucosa hiper-echoic separated by several linear tracts of contrast material, that occludes the lumen of the stomach protrudes into the liquid and antral .On this scan the length of pyloric canal and in our experience is >1.3cm mean 1.5cm.

On this view, we evaluate the “double track sign” characterized by double layer of redundant mucosa hiper-echoic separated by several linear tracts of contrast material, that occludes the lumen of the stomach protrudes into the liquid and antral . (Fig. 8) On this scan the length of pyloric canal and in our experience is >1.3cm mean 1.5cm [19].

In the our experience we evaluate 10 cases of mid-gut volvulus due a mal-rotation but we haven’t image with a whirlpool sign because the patient arrive in our emergencies room with a intestinal necrosis with the arteriovenous dilatation of the loop upstream; hyper-echoic thickening of the bowel wall edema and fluid in the intestinal tract with “tanga sign” and on the Color Doppler absence of the flow. Mal-rotation is a congenital abnormal position of the bowel within the peritoneal cavity and usually involves both the small and the large bowel [26-28]. Mal-rotation is accompanied by abnormal bowel fixation by mesenteric bands or absence of fixation of portions of the bowel, leading to increased risks of bowel obstruction, acute or chronic volvulus, and bowel necrosis. Mal-rotation occurs in approximately 1 in 500 births [29]. Mal-rotation is usually diagnosed in newborns and young infants; up to 75% of symptomatic cases occur in newborns, and up to 90% of symptomatic cases occur within the 1st year of life [29-31]. The classic clinical manifestation of mal-rotation in newborns is bilious vomiting with or without abdominal distention associated with either duodenal obstructive bands or mid-gut volvulus [31]. Mid-gut volvulus is a life-threatening condition in which the small bowel or proximal colon twists around the superior mesenteric artery (SMA) and it commonly presents during the first year of life [32-34]. Us scan and color Doppler sonography was performed in all patients suspected of having mid-gut volvulus.

The diagnosis of midgut volvulus in infants is facilitated by direct sonographic visualization of the twisted bowel loop. Normally, the SMV is on the right side of the artery. In mal-rotation, we evaluated the “whirlpool sign” on the axial scan. The whirlpool sign is formed by the mesenteric vein is on the left side of the artery [35,36].

## Conclusions

B -mode and Color Doppler ultrasonography has become the imaging modality of choice for evaluating non traumatic gastrointestinal emergencies. Ultrasound, through the research for these characteristic signs, is a valid method for the study and for the immediate diagnosis in the emergency room of these pathologies [37-39].

## Competing interests

The authors declare that they have no competing interests.

## Authors' contributions

AR wrote the manuscript. AD and AR edited early versions of the manuscript. RG also edited the final version of the manuscript, and AR adapted and revised the figures and figure legends. All authors read and approved the final manuscript.

## Declarations

This article has been published as part of *Critical Ultrasound Journal* Volume 5 Supplement 1, 2013: Topics in emergency abdominal ultrasonography. The full contents of the supplement are available online at http://www.criticalultrasoundjournal.com/supplements/5/S1. Publication of this supplement has been funded by the University of Molise, University of Siena, University of Cagliari, University of Ferrara and University of Turin.
